# The urgent need to extend the appropriate use of ultrasound in Africa and worldwide. Overview, experiences and perspectives

**DOI:** 10.3389/fpubh.2024.1363134

**Published:** 2024-06-17

**Authors:** Teresa Abbattista, Maria Franca Meloni, Giovanna Ferraioli, Clara Pirri

**Affiliations:** ^1^Accademia Ecografica Senigalliese and Senigallia Hospital, Senigallia, Italy; ^2^Valduce Hospital, Como, Italy; ^3^University of Pavia, Pavia, Italy; ^4^University of Catania, Catania, Italy

**Keywords:** ultrasound, teaching, Africa, worldwide, POCUS education

## Abstract

It is known that in African countries the health condition is problematic, both from a diagnostic and therapeutic point of view. Patients have to travel long distances to access medical care. Many cannot afford the cost of transportation to a medical facility. Ultrasound its into the scenario of healthcare imaging with limited resources, as an effective, economical, repeatable diagnostic tool, requiring low maintenance. Ultrasound tools in fact are relatively cheap and machines are easy to move, making them adapt to be taken to a rural setting where they are most needed. However ultrasound exams are not easy to perform and they need an adequate training. The spread of POCUS (point-of-care “focused” ultrasound) worldwide could be useful in Africa to identify high-risk patients. These cases selected in rural setting by POCUS can be referred to hospitals for further treatment. To deal with these situations it is necessary to form doctors and/or paramedical staff capable of guaranteeing a qualitatively adequate service. Therefore the need for basic training is greater in developing countries. Sharing successful educational strategies should advance the integration of ultrasound into the university medical school curricula. This will ensure that recently qualified doctors can practice their basic skills accurately and independently.

## Introduction

**Figure 1:**
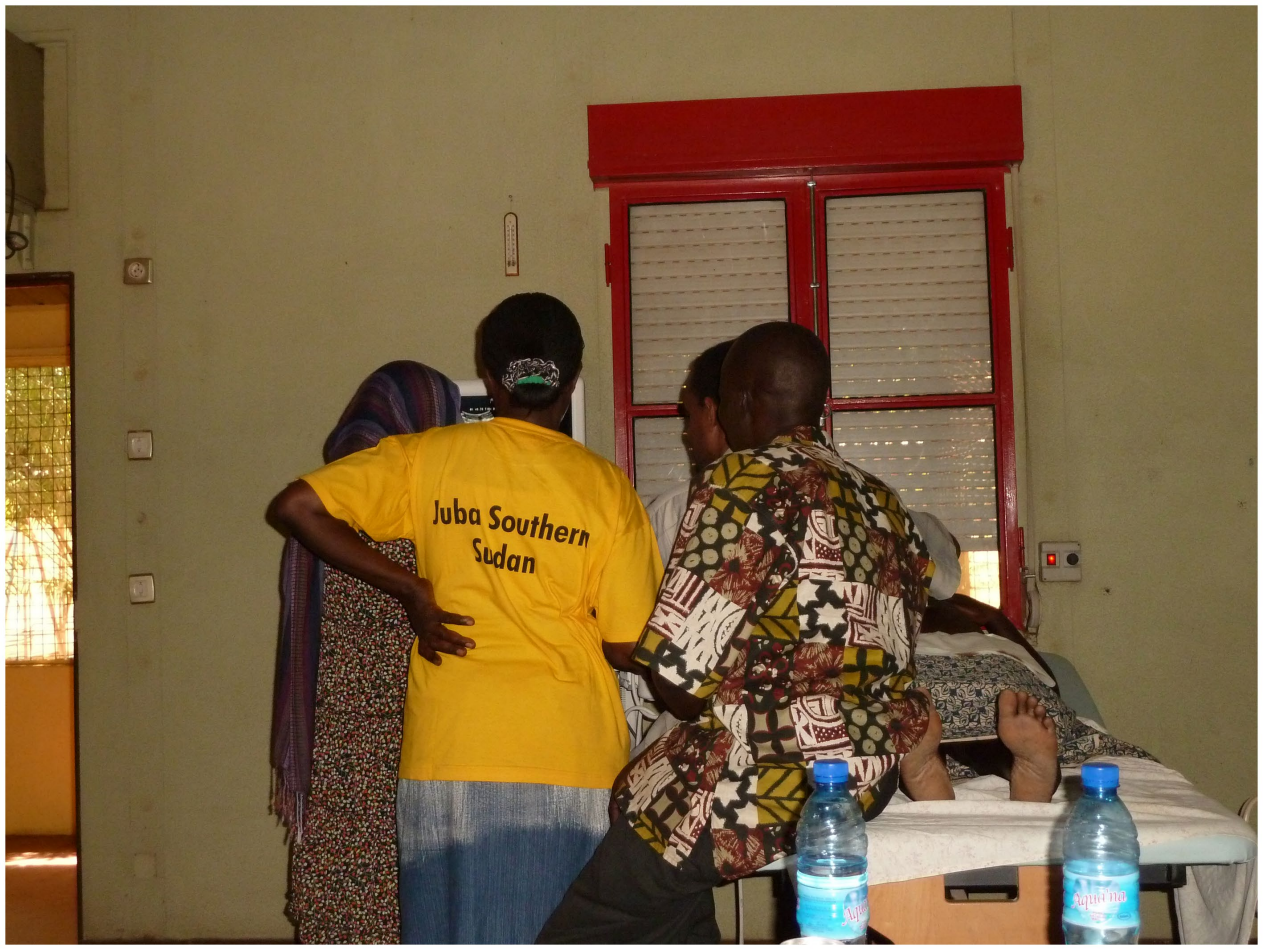
Near-peer practical lessons.

**Figure 2:**
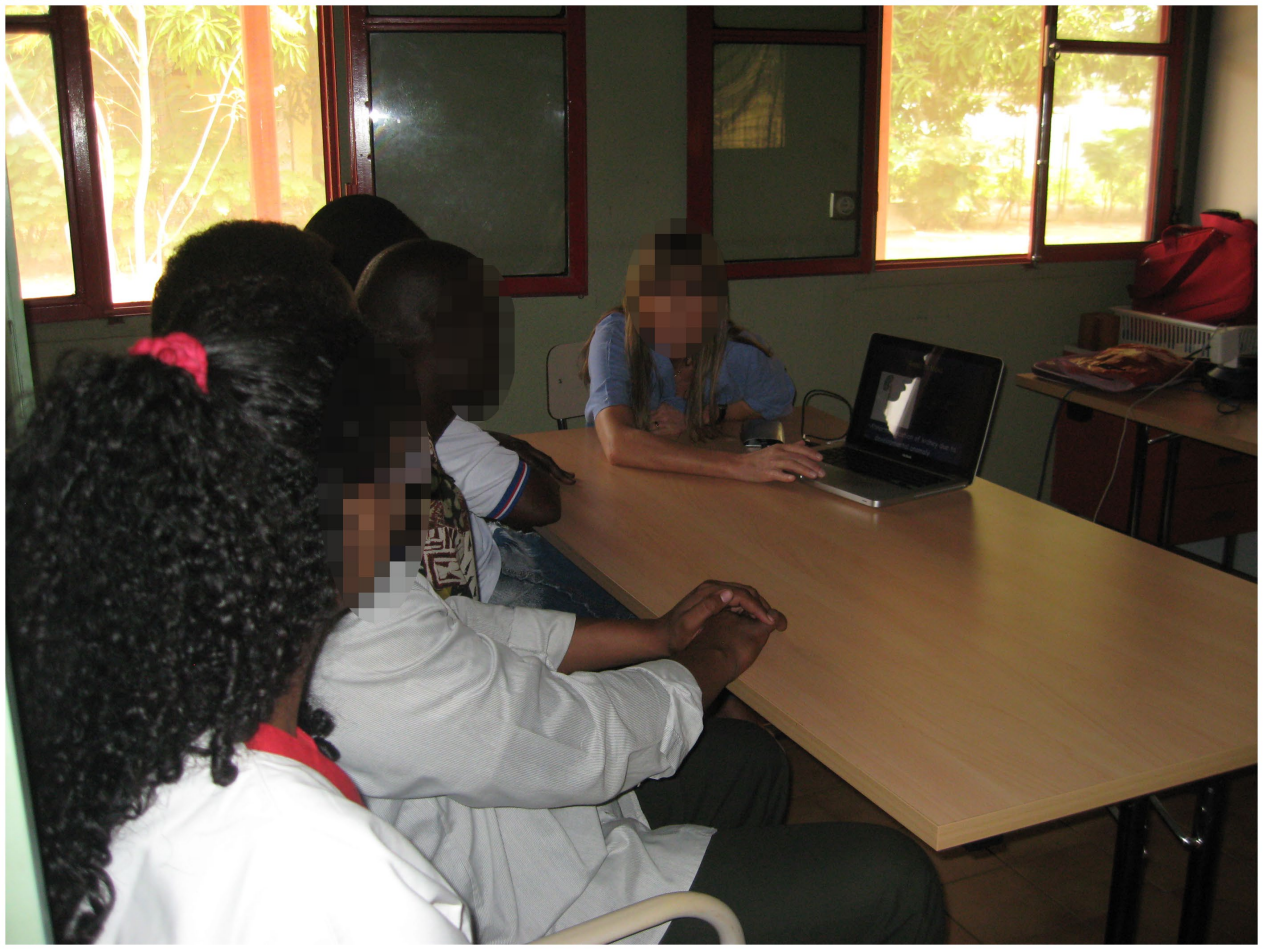
Didactic lectures.

Ultrasound is an operator-dependent technique that requires theoretical and practical know-how with intense communication skills of the operators from which considerable benefits are expected for the communities. The ultrasound imaging was diffused in medicine in the second half of the 1950s. Since its first clinical application in diagnostics, ultrasound has had a widespread and rapid diffusion with ever increasing applications in just 20 years in qualitative and quantitative terms. In the last 40 years the demand of ultrasound imaging has never stopped increasing and the ultrasound method has rapidly become the most widely used medical imaging modality worldwide for its lack of ionizing radiation, low cost and high portability (1). In most countries, the use of ultrasound was given to radiologists because they are used to working with diagnostic methods based on images. From that moment on, in many cases, they do not intend to share the ultrasound method with other specialists. Therefore, offering ultrasound to people is very limited compared to the global need. However, in recent years, many specialists have realized that it is not necessary to be a radiologist to do an ultrasound exam (2). Bedside ultrasound called Point-of-Care Ultrasound or POCUS has contributed greatly to the spread of ultrasound among non-radiologists. POCUS ultrasound is the ultrasound used to diagnose problems wherever a patient is being treated, whether in a modern hospital, an ambulance, or a remote village and it is performed usually by non-radiologists (3). Therefore, ultrasound in the hands of non-radiologists could become the turning point to multiply exponentially the supply of ultrasound exams that would spread worldwide and adapt to the most diverse diagnostic needs. In this way in poor countries and in Africa the ultrasound method could easily reach isolated areas to select patients to be centralized in the hospitals, solve problems in obstetrics or become the protagonist in war scenarios. Even in rich but sparsely populated countries and in Europe ultrasound exams made by non-radiologists would reduce the time for diagnosis as well as the efficiency in centralizing patients in hospitals (4). If radiologists would be more aware of both the current need of the use of ultrasound among non-radiologists and the current gaps of their knowledge of the ultrasound method, this would provide a solid scientific basis for proper consultation between the two. Therefore, the need of teaching ultrasound to non-radiologists is vitally important because in the face of widespread diffusion of ultrasound tools, which are relatively cheap, there is a lack of organization of ultrasound teaching methods (5). Some experiences have involved peer teaching with experienced ultrasound students teaching novice students; one of the main motivating factors for student tutors to teach was the possibility to simultaneously share and improve their knowledge and expertise (6). However a remuneration system ought to be provided for expert volunteer tutors in ultrasound who transmit knowledge of the method, thus avoiding the serious problem of lack of motivation. One example of the compensation system in the form of professional development credits given to the expert tutors in the hospitals has been already experimented in many Italian schools of ultrasound under the aegis of SIUMB (Italian Society of Ultrasound in medicine and biology) (see Figures 1, 2).

## Associations of ultrasound physicians

In recent years, doctors with various specializations interested in ultrasound have organized themselves into very large and transversal ultrasound associations worldwide. These associations follow doctors who want to learn the ultrasound method or update themselves. In particular those doctors who did not learn ultrasound during their university studies are able to attend courses which guarantee the quality of ultrasound examinations. In Europe these associations are well organized and some of them are also dedicated to teaching ultrasound in countries affected by poverty and socio-economic deprivation as well as population growth.

## WFUMB

Many ultrasound associations converge in a worldwide association named WFUMB (World Federation of Ultrasound in Medicine and Biology). WFUMB is dedicated to the advancement of ultrasound by encouraging research, promoting international cooperation, disseminating scientific information, and improving communication in the global ultrasound community. Furthermore, the mission of WFUMB is to bring sustainable ultrasound programs to the disadvantaged areas to improve global healthcare through collaboration, communication, and education (7).

## Centres of education

WFUMB has encouraged and contributed to the creation of COE (Training Centres) to provide training on ultrasound methods to post-graduate doctors worldwide (Table 1). These centres are based on the cooperation with regional ultrasound organizations and on a formal contract with the national societies for ultrasound worldwide. These centres are always dedicated to finding new ideas on how to teach ultrasound as well as innovative ways to bring ultrasound to more remote areas. The centres ought to provide certification to participants upon completion of courses as well serving as centres of reference in the regions in which they are located (8). There are some intermediary associations which collaborate with WFUMB by increasing new centres in very poor Countries. MASU (Mediterranean and African Societies of Ultrasound) is an example of these associations. MASU was composed of countries which overlook the Mediterranean basin followed quickly by many African countries (9).

**Table 1:** Countries with COE’s activity listed in WFUMB website.

Albania	Kenya	Sudan
Bangladesh	Moldova	Togo
China (Beijing)	Mongolia	Uganda
Ethiopia	Paraguay	Venezuela
Fiji	Philippines	Vietnam
Indonesia	Romania	

## Ultrasound in Africa and in developing countries

In African countries the health condition is problematic both from a diagnostic and therapeutic point of view. Ultrasound is cheap and fits in the scenario of healthcare imaging with limited resources. Technological advances now allow the possibility of better image definition and reduction of the size of the ultrasound equipment resulting in the possibility of transporting the instruments even to remote villages. Patients sometimes have to travel long distances to access medical care which many cannot afford due the cost of transportation to a medical facility (10). The role of POCUS would be to identify high-risk patients who can be referred to hospitals for further treatment. Beyond acquiring equipment, the biggest problem in these situations is the education of medical doctors and/or paramedical personnel capable of ensuring a qualitatively valid service. The extensive territorial organization in villages in Africa exacerbates the problem of basic education in developing countries compared to industrialized countries (11). The experience of World Medical Colors (WMC), an Italian association committed to the diffusion of ultrasound to doctors, is an example of cooperation between voluntary associations and local governments which has matured over 20 years. It is focused on the ultrasound training of healthcare personnel working in public facilities with limited resources and in underserved areas. The association has worked in countries such as Congo Brazzaville, South Sudan, Kenya, Zanzibar Archipelago and only in Tanzania for about 20 years. This has allowed the development of a training project over the years which has had a specific aim. Planning the education of healthcare personnel in training courses which last 3 or 5 years, depending on the background of the participants as well as the self-sustainability of the project itself are of utmost importance. It consists in identifying within the group being trained, prominent elements who can become, with their help and support, future teachers for the training of subsequent groups of healthcare workers. The result is achieving independence and consequent self-management of the project. The project is based on the selection by the local Ministry of Health regarding personnel working in public hospitals (MDs but also Assistant Medical Officers, Clinical Officers, Radiographers, and Nurses) with access to ultrasound equipment present in the hospital. After an entrance test, aimed at evaluating basic level, a maximum of 15–20 people are selected who will be included in the training project. The program also includes basic information on anatomy and pathology, when it is absent or scarce in the participants’ curriculum, in order to build a solid ultrasound knowledge. The presence of the tutors in the area is scheduled with 3 accesses per year lasting 10 days. Approximately 2, 5 h a day are dedicated to lectures and 3, 5 h are dedicated to hands-on activity. At the beginning and at the end of each session, students are subjected to entrance tests and final tests. The results of the practical ultrasound testing will allow the students to proceed to the next session. The final diploma was obtained after successfully passing the final tests of the 3–5 years of training. The first structured program began in 2006 on the island of Pemba (Zanzibar Archipelago), lasting 5 years, with exceptional results published in the American Journal of Roentgenology (12). The success achieved with this structured program was of the experience in different countries, has allowed us to understand that the project is valid, effective, repeatable and therefore worthy of being re-proposed and applied in other areas of developing countries. In 2011, in fact, in the Zanzibar Archipelago, at the Mnazi Moja Hospital (Unguja), the teaching format was applied to the second group of selected learners, introducing the new locally trained regional trainers who thus became “trainers,” with notable success in terms of outcomes both among students and in the healthcare community. The project is currently moving forward. The fourth edition of the program began in January 2023 at Mnazi-Moja Hospital with the selection of twenty participants. Over the years, many sonographers have been trained by WMC in the Zanzibar Archipelago, resulting in the performance of quality diagnostic tests confirmed by the medical, surgical and obstetric-gynaecological community, with the parallel acquisition of good quality ultrasound equipment. The Ministry of Health has recognized the importance of having efficient and effective ultrasound services and this is why the first memorandum of understanding between the WMC and the Ministry of Health was signed in 2011 (Table 2). Thanks to this work and its results, the population of the Archipelago can now carry out good quality ultrasound examinations on site without traveling to mainland Tanzania, saving time, money and discomfort for patients. Furthermore, there is an Ultrasound School in this Country which in the near future will be largely managed by local MDs and paramedical personnel, thus achieving the aim of the self-sustainability of the project. This training format developed by WMC can also be exported and applied in other developing countries (13). In Africa there are only a few developed radiological services that perform ultrasound scans. To overcome dependence on voluntary associations for teaching ultrasound, the introduction of ultrasound at university level would bring multiple advantages for the training of doctors and healthcare workers. The advantages range from a teaching tool, particularly for teaching anatomy in “real-time,” to the acquisition of skills to perform ultrasound examinations after graduation. This could be pursued in the future with POCUS. Unfortunately, at present, there are only a few universities in Africa that have adopted or intend to adopt the ultrasound method. However, the basic elements common to all curricula, regardless of region, are understanding the physics/functioning of the ultrasound machine and good governance in ultrasound practice. The methodology of the study program delivery centres on the various stages of training may vary depending on local conditions. There is evidence to suggest that competence can be presumed after a relatively small number of ultrasound examinations have been performed. Some experiences have revealed that for obstetric scanning a one-month training period is sufficient to initiate a skills assessment. One area of medicine where POCUS is highly applicable is prenatal care. Very few women in sub-Saharan Africa have access to antenatal care and the maternal mortality rate is unacceptably high. Pilot projects in Kenya have found that midwives can be trained to perform basic ultrasound scans to identify high-risk pregnancies. The training period is 1 month and the accuracy of midwives in identifying high-risk pregnancies is very high. Using portable, battery-powered machines, midwives can scan patients in their homes and villages. Images and provisional reports can be transmitted to a specialist using mobile phone technology and simple modems. Image transmission times are low and the image is without loss. Since there is a shortage of specialists, this type of teleradiology is extremely useful. All high-risk pregnancies can be referred to specialized centres, thus reducing the possibility of adverse outcomes (14–16) (Table 3). There is wide variability in the ultrasound training of medical students around the world. Africa, the continent with the greatest health challenges, has yet to fully utilize this important technology. The need for this technology is great and the conditions requiring its application are increasing. The availability of structured training programs during medical school is still rare and many doctors still acquire all their ultrasound skills during postgraduate training. Anecdotal evidence is positive and this is supported by publications from developing countries (17).

**Table 2:** Zanzibar project 2006–2010 – Chake Chake Hospital.

10 trainees (1 physician, 7 radiographers and 2 assistant medical officers)
European physicians teachers.
Courses every 4 months for 2 consecutive weeks each time. During each course, 24 h of didactic lectures and 36 h of practice were planned
Machine: Esaote Biomedica AU 450 unit equipped with a 3.5-MHz convex probe
1th year anatomy of the major organs
2nd year anatomy and pathology basics
3rd year more advanced pathologies
4th year specialized courses
5th year interactive lectures
Project cost: 50000 Euros

**Table 3:** Frequent pathologies that benefit from ultrasound in Africa.

Pathologies	Wanted signs
Tuberculosis	Extrapulmonary tuberculosis
Acute heart failure	Pericardial effusion
Maternal and neonatal problems	Neglected uterine fibroids, mola, pregnancy ovarian tumors fetal malpresentation, multiple gestations, ectopic pregnancy, and placenta previa
Pediatric pathologies	Pediatric acute care setting

## Ultrasound way of teaching in the USA

In the USA, we have witnessed one of the most interesting experiences in the teaching of ultrasound. In 2011, in fact, the School of Medicine of the University of South Carolina created the most advanced medical school program in the world (18). They have integrated an ultrasound program for all students, throughout all 4 years of medical school. The student ultrasound program was based on POCUS ultrasound originally developed for postgraduate emergency medicine physicians. Subsequently, the clinical use of ultrasound at the patient’s bedside entered clinical practice, revolutionizing the daily life of non-radiologists. As a result, the United States has achieved the greatest integration of ultrasound into the medical school curriculum. The doctors can perform ultrasound examinations at the bedside of the patients. This enables doctors to understand the ultrasound images and give assistance with immediate diagnostic decisions as well as guiding procedures such as vascular access. However, POCUS has controversial value in the world: some educators have expressed concern that teaching medical students to use ultrasound will lead to degradation of time – honored physical examination skills. However, POCUS is not intended to replace all aspects of the physical exam. Like any properly used investigation, it provides additional information that complements the physical examination, allowing for quicker and more accurate assessments, and is particularly useful where the physical examination is known to be difficult or imprecise. Numerous medical schools in different parts of the world now incorporate ultrasound training into their curriculum, ranging from adjunctive teaching to full integration into the course as a clinical tool. After this experience, SUSME, the medical training company in ultrasound, was created in the United States. Its purpose is to identify the best way to teach ultrasound in US universities and for all specialists to annually update the guidelines for the correct use of ultrasound (19). This experience could be the meeting point between the global north and south regarding ultrasound in the introduction of such ultrasound programs in universities around the world. The use and teaching of ultrasound differ from continent to continent and country to country. Great attention is given to the different uses of POCUS and their clinical significance, particularly in the fields of emergency medicine, intensive care, cardiology, anesthesiology, rheumatology, obstetrics, neonatology, gynecology, gastroenterology and many other applications (20–22). The long experience of the ultrasound method in the Global North suggests that ultrasound is a complex skill to learn, which includes the ability to obtain images through the movement of the probe and the knowledge to interpret the images in order to be able to give overall clinical judgment to manage patient problems. This method requires complex visual perception and psychomotor skills, as well as customized periods of hands-on training (23).

## Ethics statement

Written informed consent was obtained from the individual(s) for the publication of any identifiable images or data included in this article.

## Author contributions

TA: Writing – original draft, Writing – review & editing. MM: Writing – original draft, Writing – review & editing. GF: Writing – original draft, Writing – review & editing. CP: Writing – original draft, Writing – review & editing.
